# Reversal of doxorubicin resistance in lung cancer cells by neferine is explained by nuclear factor erythroid-derived 2-like 2 mediated lung resistance protein down regulation

**DOI:** 10.20517/cdr.2019.115

**Published:** 2020-04-17

**Authors:** Poornima Paramasivan, Jothi Dinesh Kumar, Rathinasamy Baskaran, Ching Feng Weng, Viswanadha Vijaya Padma

**Affiliations:** ^1^Department of Biotechnology, School of Biotechnology and Genetic Engineering, Bharathiar University, Coimbatore, Tamil Nadu 641046, India.; ^2^Department of Cellular and Molecular Physiology, Institute of Translational Medicine, University of Liverpool, Liverpool L3 5TR, UK.; ^3^Laboratory of Molecular Physiology, Institute of Biotechnology, Department of Life Sciences, National Dong Hwa University, Hualien 974, Taiwan.; ^4^Division of Science, School of Applied Sciences, University of Abertay Dundee, Dundee DD1 1HG, UK.; ^5^Department of Bioinformatics and Medical Engineering, Asia University, Taichung 41354, Taiwan.

**Keywords:** Doxorubicin, neferine, reactive oxygen species, lung resistance protein, nuclear factor erythroid-derived 2-like 2, multidrug resistance

## Abstract

**Aim:** Development of multi drug resistance and dose limiting cardiotoxicity are hindering the use of Doxorubicin (Dox) in clinical settings. Augmented dox efflux induced by lung resistance protein (LRP) over expression has been related to multi drug resistance phenotype in various cancers. An alkaloid from lotus, Neferine (Nef) shows both anticancer and cardioprotective effects. Here, we have investigated the interconnection between nuclear factor erythroid-derived 2-like 2 (NRF2) and LRP in Dox resistance and how Nef can overcome Dox resistance in lung cancer cells by altering this signaling.

**Methods:** Anti-proliferative and apoptotic-inducing effects of Nef and Dox combination in Parental and Dox resistant lung cancer cells were determined in monolayers and 3D spheroids. Intracellular Dox was analyzed using flow cytometry, siRNA knockdown and western blot analysis were used to elucidate NRF2-LRP crosstalk mechanism.

**Results:** We observed that the Dox resistant lung cancer cells expressed higher levels of LRP, reduced glutathione (GSH) and NRF2. Combination of Dox and Nef induced apoptosis, leads to reactive oxygen species (ROS) generation, GSH depletion and reduction in LRP levels contributing to higher intracellular and intranuclear Dox accumulation. The use of N-acetylcysteine and knockdown studies confirmed an important role of ROS and NRF2 in LRP down regulation. Presence of NRF2 binding sites in LRP is support of direct interaction between LRP and NRF2.

**Conclusion:** Nef sensitizes lung cancer cells to Dox by increasing intracellular and/or intra nuclear Dox accumulation via LRP down regulation. This is mediated by redox regulating NRF2. This decoded crosstalk mechanism reinforces the role of NRF2 and LRP in Dox resistance and as an important anticancer target.

## Introduction

Lung cancer is the leading cause of mortality worldwide. Postoperative chemotherapy is an important adjuvant treatment in non-small cell lung cancer (NSCLC). Despite best treatment efforts, tumor recurrence often occurs after chemotherapy due to multidrug resistance, and thus is a major impediment in lung cancer management^[1-3]^. The anthracycline antitumor antibiotic Doxorubicin (Dox) is an FDA approved chemotherapeutic drug commonly used to treat various cancers including lung cancer. There are 2 major mechanisms of action of Dox: (1) intercalation into DNA and disruption of topoisomerase-II-mediated DNA repair; and (2) generation of free radicals and their damage to cellular membranes, DNA and proteins^[4]^. From its discovery and introduction in several investigational and approved chemotherapy regimens, Dox has contributed to improved life expectancy of countless cancer patients^[5]^. However, the clinical efficacy and usefulness of Dox-based treatment regimens is still limited because of dose-limiting toxicity and induction of drug-resistance overtime^[6]^. In advanced NSCLCs, Dox treatment provides only an overall response rate of 30%-50% and most of the patients develop resistance towards Dox treatment^[7]^.

Multi-drug resistance (MDR) is a phenomenon whereby cancer cells are unaffected by various anticancer drugs that are structurally and functionally different from the initial chemotherapy^[8]^. This is the main hurdle to achieving successful chemotherapy. Mechanistically, the resistance phenomena may be explained by alteration in membrane transport proteins to increase drug efflux, enhancing DNA repair, modifying cell cycle regulation to block apoptosis, and detoxification^[9-11]^. Various membrane transporters have been implicated in drug resistance, however ABCB1 (MDR-1, P-gp), ABCC1 (MRP1) and ABCG2 (BCRP) have been most extensively studied^[12]^. Lung resistance protein (LRP), a 110-kDa vesicular protein is identical to human major vault protein, and is associated with resistance to anticancer drugs including Dox, etoposide, paclitaxel, cisplatin and carboplatin^[13-15]^. Vaults have been connected with vesicular and nucleocytoplasmic drug transport based on their subcellular localization. LRP overexpression was originally found in Dox resistant NSCLC cell line and subsequently detected in other cell lines of different histogenetic origin^[16-18]^. The lower sensitivity of non-small cell lung cancer (A549) cells than breast cancer (MCF7) cells to Dox was found to be predominantly due to the high intracellular expression of LRP than P-gp and MRP1^[17]^.

Nuclear factor erythroid-2 related factor 2 (NRF2), a redox-sensitive transcription factor, regulates cellular defense response through antioxidant response elements which confer cytoprotection against oxidative stress and apoptosis. On the dark side, constitutive activation of NRF2 contributes to chemoresistance by upregulation of glutathione, thioredoxin and drug efﬂux pathways in lung cancer cells^[19]^. Previously, it has been reported that multidrug resistance proteins MRP/ABCC 1,2,3,4,5 and breast cancer resistance protein (BCRP/ABCG2) are regulated by NRF2 mediated antioxidant response element - driven transcription in lung cancer^[20-24]^. Expression and transport functions of P-gp, MRP2 and BCRP are reported to be upregulated with NRF2 activation in blood-brain and blood-spinal cord barriers^[25]^. With increasing interest in the role of NRF2 in chemoresistance, we attempted to investigate whether LRP is also regulated by NRF2.

The combined regimen with drug efflux pump inhibitors including cyclosporin A, ketoconazole, and verapamil increased the toxic side effects associated with Dox treatment, thus decreasing the quality of life of cancer patients^[26]^. Therefore, it is necessary to use Dox in combination with an agent that abrogates Dox resistance by curtailing its toxicity. In an attempt to abate the side-effects and enhance the clinical efficacy of Dox, many plant derivatives have been used with varying degrees of success. Here, we hypothesized that Neferine (Nef) derived from the seed embryo of lotus (*Nelumbo nucifera*) can enhance the clinical efficacy of Dox without causing any side-effects*.* The role of Nef as a chemopreventive agent has been emphasized, it inhibits angiotensin II stimulated vascular smooth-muscle proliferation, induces reactive oxygen species (ROS)-dependent mitochondrial mediated apoptosis in liver and lung cancer cells, inhibits the proliferation of osteosarcoma cells and inhibits growth and migration of gastrointestinal stromal cells^[27-31]^. Nef could enhance the cytotoxicity of anticancer drugs and reverse the multidrug-resistance in cancer cells by down regulating P-gp and/or MRP1^[32-37]^. Nef also exhibits protective effects against drug/hypoxia induced cardiotoxicity^[38-42]^, which is very common in patients on a Dox regimen.

The scope of the present study is based on the assumption that LRP expression in both Dox sensitive and resistant A549 cells is regulated by NRF2, thereby oxidative stress. We have tested whether Nef can abrogate Dox resistance and enhance cancer cell response to treatment with Dox. We provide evidence that Nef could effectively reverse Dox resistance of lung cancer cells by NRF2 mediated LRP downregulation, thereby increasing Dox intracellular accumulation. Our results were also extrapolated to 3D spheroids where combined regimen of Nef and Dox could lead to significant spheroid shrinkage and cell death. As a derivative of staple food, we also expect that Nef may confer health benefits to cancer patients and thus protect them against Dox-induced cardiotoxicity.

## Methods

### Chemicals

Nef, Dox, 3-(4,5-Dimethylthiazol-2-Yl)-2,5-Diphenyltetrazolium Bromide (MTT), Cell Counting Kit-8 (CCK-8),2’,7’-dichlorodihydrofluorescein diacetate (H2DCFDA), 3,3′-dihexyloxacarbocyanine iodide (DiOC6) were obtained from Sigma-Aldrich. DMEM, FBS and all other cell-culture reagents were obtained from Hi-media Laboratories, India. Reagents for assays were obtained from Merck specialty Chemicals, India. All primary antibodies used were obtained from Cell signaling technology, USA and Upstate, USA. HRP-conjugated secondary antibodies, was purchased from Leinco Technologies, USA. Western-blot membranes were obtained from Whatman, USA.

### Cell culture

Lung cancer cells, A549 were obtained from National Centre for Cell Science (NCCS), Pune, India. The Dox resistant counterparts A549/Dox were developed by continuous exposure to increasing concentrations of Dox. Dox resistance was maintained via selective pressure by culturing cells in a medium supplemented with 0.5 μM Dox. Drug resistance was verified every 3 months against the parental cells by MTT assay. All the cells were grown in DMEM and 10% FBS (v/v), containing 100 units/mL penicillin, 30 µg/mL streptomycin and 20 µg/mL gentamycin in a 37 °C incubator with 5% CO_2_.

### Cell viability assay

The effects of Nef on the cytotoxic potential of Dox were assessed using MTT. The results were used to calculate combination index (CI) and to plot isobologram.

### Establishment of 3D spheroids and viability assay

Cells were seeded at a density of 2500 cells/well in ultra-low attachment 96 well round bottom plate and allowed for multicellular spheroid formation for a week. Spheroids were subsequently treated with Dox and Nef alone or in combination for 48 h. Spheroid formation and growth was assessed via microscopic examination using an inverted microscope, the images were analyzed by Image J software and the cell viability was assessed using CCK-8.

### Apoptosis measurement by flow cytometry

Cancer cells in monolayer or spheroids were exposed to Dox alone or in combination with Nef. After treatment for 48 h, cells were trypsinized and centrifuged and the pellet washed twice with PBS. Cells were resuspended and then washed the cells with PBS three times. Apoptotic cells were detected with Annexin V-FITC/PI according to the protocol of Annexin V-FITC cell Apoptosis Detection Kit (BD, USA).

### Intra-cellular and nuclear Dox accumulation

Cells were exposed to Dox for 4 h with or without Nef and then washed three times with ice-cold PBS. Cells were lysed with PBS containing 0.1% triton X-100 and intracellular accumulation of Dox was measured by flow cytometry or fluorescent plate reader, using excitation and emission wavelengths of fluorescence 485 nm and 590 nm, respectively. To analyze the Dox accumulation in nucleus, the nuclear fraction was separated using nuclear fractionation buffer and followed the same procedure as for total cell lysate. The intensity emitted was translated into concentrations of drug using a Dox standard curve and expressed as nM Dox in the cells assessed before and after treatment. The cells were grown on coverslips and treated with Nef and Dox for fluorescent microscopic analysis.

### siRNA transfection

Cells were reverse transfected with 10 nM of LRP siRNA or NRF2 siRNA from Santa Cruz Biotechnology using Interferin reagent (Polyplus transfection Inc., USA) according to manufacturer’s protocol and processed 48 h after transfection. Knockdown was confirmed by western blotting and the concentration of the siRNA did not affect the cell viability.

### Western blot analysis

Cells were collected and washed twice in PBS, then lysed in ice-cold lysis buffer (50 mM Tris-HCl, pH-7.4, 150 mM NaCl, 5 mM EDTA, 50 mM NaF, 1% Triton X-100, 1 mM sodium-orthovanadate, 1 mM phenylmethanesulfonylﬂuoride, 1 mg/mL aprotinin, 2 µg/mL pepstatin-A, and 2 µg/mL leupeptin) on ice for 1 h. Cell lysates were then centrifuged for 15 min at 13,000 rpm at 4 °C. Proteins were separated using SDS-PAGE and transferred to PVDF membrane. The blots were blocked with 5% non-fat milk in TBST at RT for 1 h and incubated overnight with the appropriate primary antibody at 4 °C. After wash, the blots were incubated with peroxidase-conjugated secondary antibody for 1 h. Bands were monitored using Western-blot chemiluminescence reagent (Amersham Biosciences Corp., USA). β-actin/GAPDH were used as an internal control. Densitometric-analysis was carried out using multiguage image digitalizing software.

### ROS and reduced glutathione measurement

Relative changes in intracellular reactive oxygen species in A549 cells were monitored using a fluorescent probe, 2’,7’-dichlorofluorescein diacetate (DCFH-DA). Cells were seeded at a density of 1 × 10^5^ cells/well. Cells were incubated with 10 μM of DCFH-DA and incubated for 30 min, followed by incubation with different concentrations of Nef and Dox for different time periods (15 min, 30 min and 1 h). One treatment group with 50 μM H_2_O_2_ was included to serve as a positive control. Then the cells were harvested, centrifuged, washed and re-suspended in PBS and read in a Hitachi spectro-fluorimeter using excitation/emission as 480 nm/520 nm. The values were expressed as % relative fluorescence as compared to the control.

The intracellular concentration of reduced glutathione was estimated by the fluorometric assay as described by Pereira-Caro *et al.*^[43]^, with slight modifications. Cells from the different treatment groups were treated with 25% orthophosphoric acid followed by sonication. The samples were centrifuged to precipitate proteins and the supernatant was suitably diluted in 0.1 M phosphate EDTA buffer, pH 8.0. Ten μL of ortho-pthalaldehyde (5 mg/mL) was added and incubated for 10 min in the dark. The samples were then analyzed in a fluorimeter at excitation 340 nm and emission 460 nm. The values obtained were calculated based on a glutathione standard graph in the linear region using regression analysis and were expressed as μmoles of reduced glutathione (GSH)/mg of protein.

### Measurement of mitochondrial membrane potential

Treated and untreated cells were exposed to 50 nM [DiOC_6_ (3)] at 37 °C for 30 min. The cells were collected, resuspended in 2 mL PBS after washing twice with PBS and fluorescence intensity was measured in a fluorescent spectrophotometer using Excitation/Emission as 488 nm/500 nm.

### Statistical analysis

All experiments were performed three times in triplicates. Results were expressed as mean ± SEM. The statistical analysis was carried out using one way ANOVA followed by Tukey’s test. Differences were considered statistically significant when *P* < 0.01 (**) or *P* < 0.05 (***).

## Results

### Nef augments the anti-proliferative activity of Dox

**Figure 1:**
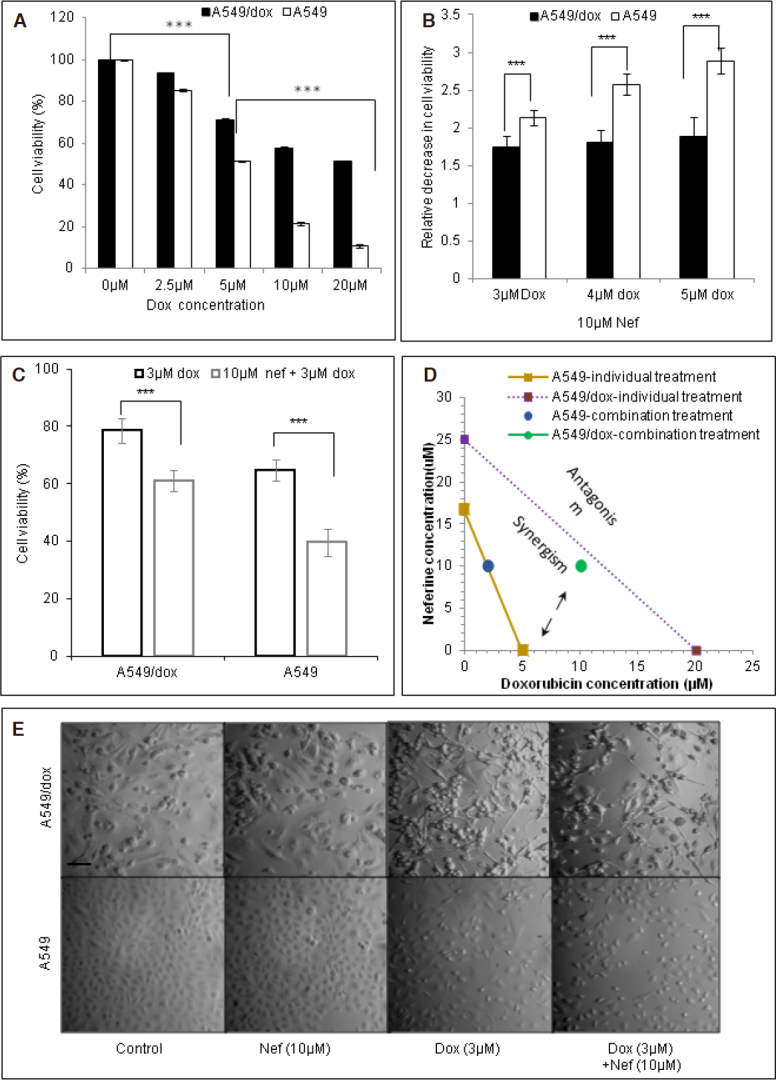
Nef augments the antiproliferative activity of Dox. Bar graph representing the inhibition of A549 and A549/Dox cell viability using: A: Dox alone; B: combination of Nef and various concentrations of Dox administrated at the same time for 48 h; C: comparing the cytotoxic effect of Dox alone and Nef and Dox in combination; D: normalized isobologram showing additive/synergistic interaction between Nef and Dox. Each bar represents the mean ± SEM of 3 experiments and *** represents *P* < 0.01 or ** represents *P* < 0.05; E: photomicrograph of cultured A549 and A549/Dox cells comparing the cytomorphology and post-treatment morphologic changes after Dox only or Nef and Dox combined treatment (Magnification 100×). Nef: Neferine; Dox: Doxorubicin

A549 cells that harbor point mutation in the Keap1 gene (*G333C*) and loss of heterozygosity at 19p13.2 that leads to the loss of KEAP1 activity and gain of NRF2 function^[44]^ were chosen for the study. A549/dox cells have been developed by continuous exposure of increasing concentrations of Dox for 6 months. Exposure of various concentrations of Dox induced A549 and A549/Dox cell death in a concentration dependent manner. The developed A549/Dox cells were 4 times resistant than the parental A549 cells [Figure 1A]. Treatment with Dox not only leads to the primary resistance but also in cross resistance to other anticancer compounds such as paclitaxel and cisplatin. The developed A549/Dox cells had higher levels of P-gp, LRP, MRP1, BCRP, NRF2 and GSH than the A549 cells [Supplementary Figure 1A-C]. Nef concentration was chosen based on the results of previous studies^[30]^. Dox at 3 μM, 4 μM and 5 μM concentrations showed 2.13, 2.57 and 2.88-fold decrease in cell viability of A549 cells and 1.73, 1.80 and 1.88-fold decrease in cell viability of A549/Dox cells with simultaneous treatment of Nef and Dox in comparison with cells exposed to Dox alone [Figure 1B]. Simultaneous exposure of A549 and A549/Dox cells to Dox in presence of 10 μM Nef for 48 h significantly increased the sensitivity of these cells to Dox compared to Dox treatment alone [Figure 1C]. The effects of pre-treatment/post-treatment (24 h/24 h) of Nef on percentage cell viability were also analyzed which were less significant than the simultaneous treatment (Data not shown). Hence further studies were carried out following the simultaneous treatment system. The combined regimen of Nef and Dox could effectively reduce the number of colonies formed than the individual treatments [Supplementary Figure 1D]. The combinatorial drug interaction index (CI) analysis revealed that the effect of Nef and Dox together on A549 cells (CI = 1.0) was additive and A549/Dox cells (CI = 0.75) were synergism [Figure 1D]. Dox resistant cells showed a different morphology than the parental cells. Morphological changes after the Dox and Nef treatment were observed using light microscopy and depicted in Figure 1E.

### Induction of apoptosis in Nef and Dox treated cells

**Figure 2:**
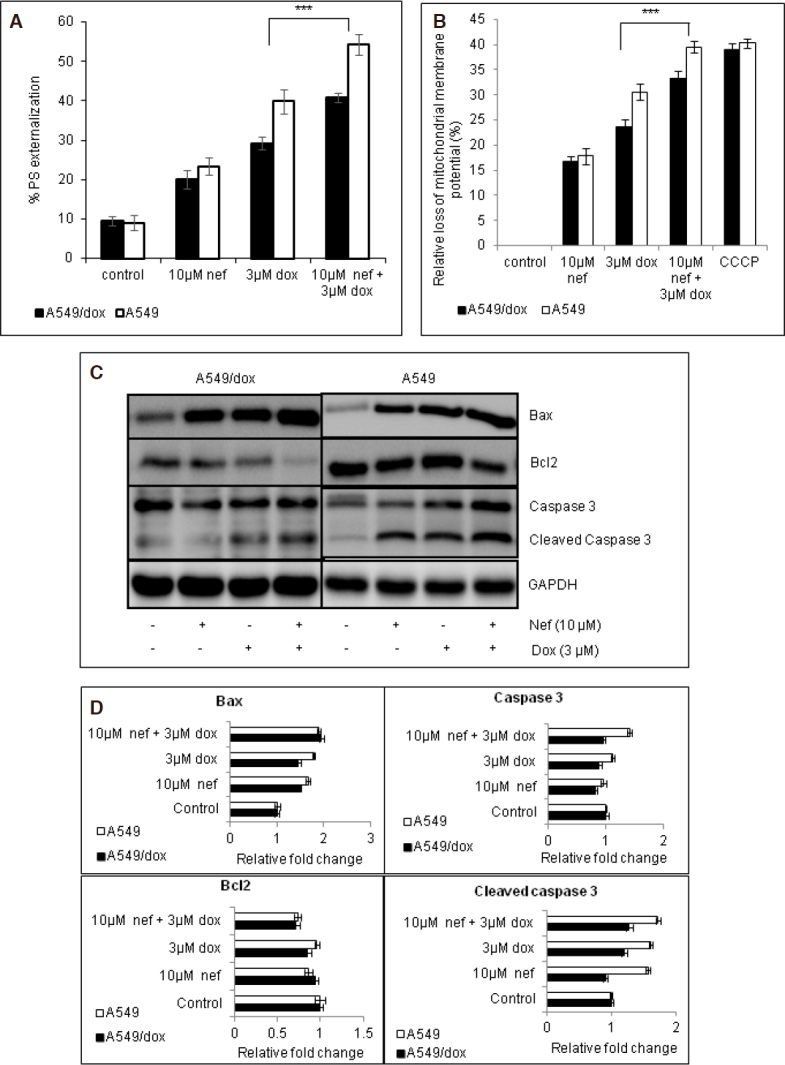
Nef-Dox combination therapy induces apoptosis in treated lung cancer cells. Line graph showing the percentage (%) of: A: phosphatidyserine externalization using flow cytometric analysis; B: loss of mitochondrial membrane potential using fluorescence spectrophotometer; C, D: representative western blot analysis of A549 and A549/Dox cells treated with Dox alone, Nef alone and Nef and Dox combination and densitometric analysis are shown. Bax, Bcl2, caspase 3 and cleaved caspase 3 protein expressions were probed. GAPDH served as the loading control. PS: phosphatidylserine ; Nef: Neferine; Dox: Doxorubicin; GAPDH: Glyceraldehyde 3-phosphate dehydrogenase

To determine the apoptotic inducing potential of the combination treatment, cells were analyzed for apoptotic induction as percentage of phosphatidyl serine (PS) externalization and mitochondrial membrane potential (DYM). Cells treated with Dox did show slight increase in %PS externalization and mitochondrial membrane potential in A549/Dox cells compared to control whereas lower than the A549. Of importance is that the percentage of increase was significantly high in the cells which received Nef and Dox in combination [Figure 2A and B]. To correlate with the higher %PS externalization, the expression levels of Bax, Bcl2 and cleaved caspase 3 were analyzed by western blot. The expression of Bax, and cleaved caspase 3 were higher with a decline of Bcl2 in the cells treated with Nef and Dox in combination than the cells treated separately [Figure 2C and D]. Bax/Bcl2 ratio was found to be high in the cells received combination of Nef and Dox than the cells treated with Dox alone [Supplementary Figure 1E]. This confirmed that the apoptotic inducing potential of combined regimen of Nef and Dox is higher than the Dox alone in all the tested cell lines.

### Nef and Dox enhances intracellular Dox accumulation by LRP downregulation

**Figure 3:**
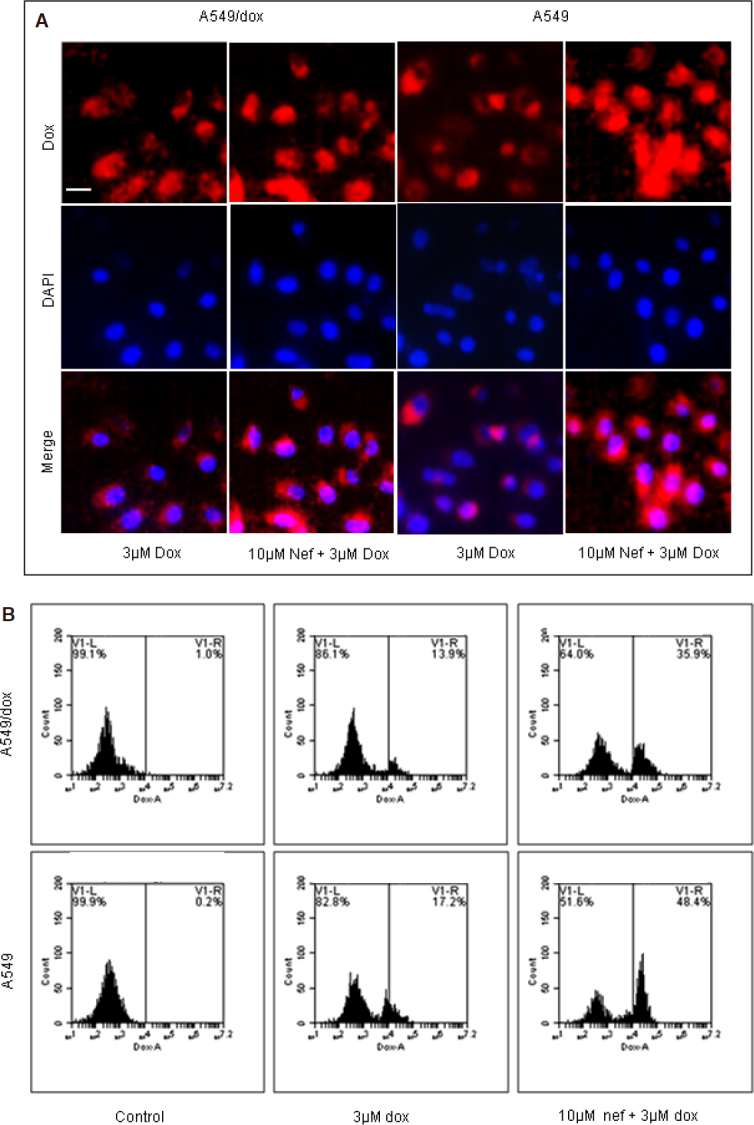
Intracellular DOX accumulation was potentiated by Nef. A549 or A549/Dox cells were treated with 10 μM Nef and/or 3 μM Dox and the intracellular dox was assessed as mentioned in materials and methods: A: fluorescent microscope (magnification 400×); B: flow cytometry. Results shown are are three separate experiments performed in triplicate. Nef: Neferine; Dox: Doxorubicin

**Figure 4:**
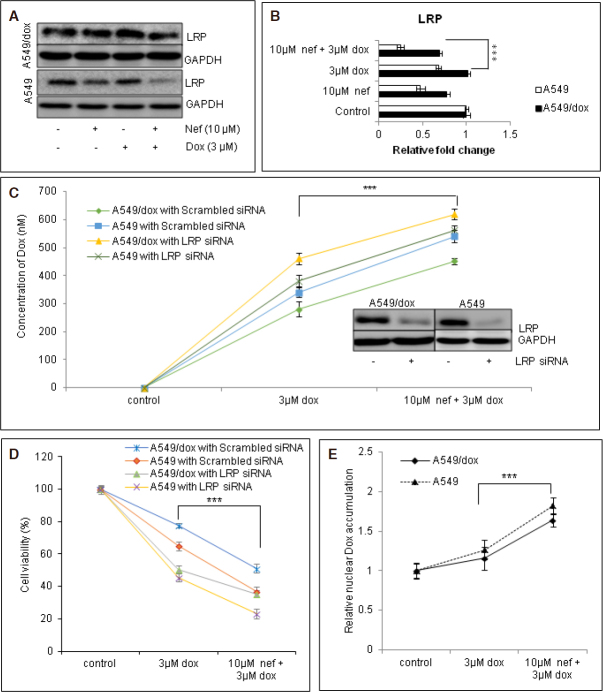
Nef increases intra-cellular and intra-nuclear Dox by LRP downregulation. A549 or A549/Dox cells were treated with 10 μM Nef and/or 3 μM Dox and (A) and (B). Analysed for LRP expression, representative western blot image of A549 and A549/Dox cells treated with Dox alone, Nef alone and Nef and Dox combination and densitometric analysis are shown. A549 or A549/Dox cells were knocked out for LRP using siLRP and treated with Dox alone or in combination with Nef; C: the intracellular Dox was assessed and the results were expressed as nmoles of Dox; D: cell vaibility was assessed; and E: intranuclear Dox accumulation was assessed. The results shown are mean ± SEM and *** represents *P* < 0.01 or ** represents *P* < 0.05, which are three separate experiments performed in triplicate. Nef: Neferine; Dox: Doxorubicin; LRP: lung resistance protein; siLRP: small interfering LRP; GAPDH: Glyceraldehyde 3-phosphate dehydrogenase

The intracellular concentration of Dox was assessed in the presence or absence of Nef. Our data suggested that Nef treatment significantly increased the Dox accumulation in lung cancer cells [Figure 3A and B, [Supplementary Figure 1E]. There are reports suggesting that the least sensitivity of lung cancer cells to Dox were due to the sequestration of Dox in cytoplasmic compartments by LRP than the breast cancer cells^[17]^. Hence, we were interested to study the levels of LRP in Dox sensitive and resistant lung cancer cells. We found that the LRP levels in the A549/Dox cells are significantly higher than in A549 cells. Nef could downregulate the expression of LRP in both the cell lines whereas the downregulation by Nef and Dox combined regimen was significant compared to Dox alone treated cells [Figure 4A and B]. Knockdown of LRP by siRNA lead to the significant increase in combined regimen induced intracellular Dox accumulation [Figure 4C] and cell death [Figure 4D]. These results confirm the role of LRP in Nef and Dox combination mediated reversal of Dox resistance in lung cancer cells.

### LRP down regulation increases intra-nuclear Dox

LRP has been reported to play vital role in transport of Dox from nucleus to the cytoplasm^[45]^. Hence, we presumed that there may be difference in the nuclear Dox levels. To confirm this presumption, we analyzed the nuclear levels of Dox in cancer cells in the presence or absence of Nef. As anticipated, the Dox levels in the nuclear fraction of Nef and Dox treated cells were significantly higher than the Dox alone treated cells [Figure 4E]. Thus, the results of our study evidence the intra nuclear accumulation of Dox by Nef and Dox simultaneous treatment in parental and Dox resistant lung cancer cells.

### LRP down regulation by Nef and Dox combination is mediated by ROS

**Figure 5:**
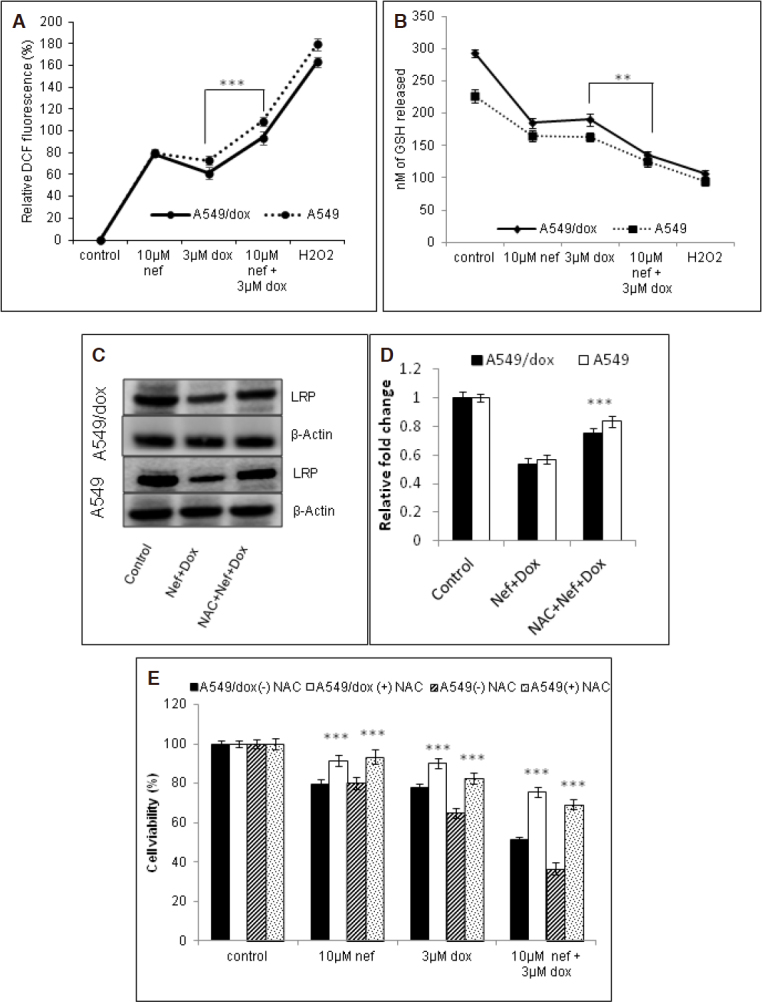
Nef and Dox combination induced ROS mediated LRP down regulation. A549 and A549/Dox cells with Dox or Nef alone or their combination: A: intracellular ROS concentration was determined using DCF; B: intracellular GSH was assessed using OPT and the fluorescence was measured by fluorometry as explained under Materials and methods. 50 µM H2O2 was used as positive control. The results shown are mean ± SEM and *** represents *P* < 0.01 or ** represents *P* < 0.05, which are three separate experiments performed in triplicate. A549 and A549/Dox cells treated with Dox/Nef alone, NAC alone and NAC and Nef + Dox combination; C, D : the cell lysate was analysed for LRP expression, a representative image of three experiments and densitometric analysis are shown. GAPDH served as loading control and (E) the cell viability was assessed, the results shown are mean ± SEM and *** represents *P* < 0.01 which are 3 separate experiments performed in triplicate. Nef: Neferine; Dox: Doxorubicin; LRP: lung resistance protein; NAC: N-acetyl cysteine; OPT: ortho-pthalaldehyde; GAPDH: Glyceraldehyde 3-phosphate dehydrogenase; ROS: Reactive oxygen species; GSH: Reduced glutathione; DCF: 2’, 7’ –dichlorofluorescein

Previously we have reported that Nef increases intracellular ROS and depletes intracellular antioxidant levels^[30]^. We assumed that Nef induced ROS levels has direct effect on LRP function. To substantiate this assumption, in the first set of experiments we examined ROS levels in the presence and absence of Dox and/or Nef. Furthermore, the ROS levels in the presence of H_2_O_2_ (positive control) were also analyzed. Nef cotreatment led to significant increases in ROS levels compared to Dox treatment alone. To maintain cellular homeostasis, ROS levels were counterbalanced by intracellular antioxidants. Hence, the intracellular level of cellular antioxidant GSH was measured. Our data suggested that the intracellular ROS levels were inversely correlated with GSH content in the cancer cells when treated with Nef and Dox [Figure 5A and B]. As expected, ROS levels were not significantly different from control when the cells were treated with antioxidant *N*-acetyl cysteine (NAC). Furthermore, LRP level in the presence of NAC was found to be higher than in the absence of NAC [Figure 5C and D]. This confirms that the LRP downregulation by combined regimen of Nef and Dox is mediated by ROS. In addition, cell viability of the cells received Nef and Dox in the presence of NAC were significantly higher compared to the cells treated in the absence of NAC [Figure 5E]. The data from our study exemplifies the ROS mediated LRP downregulation and cell death induction by Nef and Dox in A549 and A549/Dox cells.

### Role of NRF2 in reversal of Dox resistance by Nef

**Figure 6:**
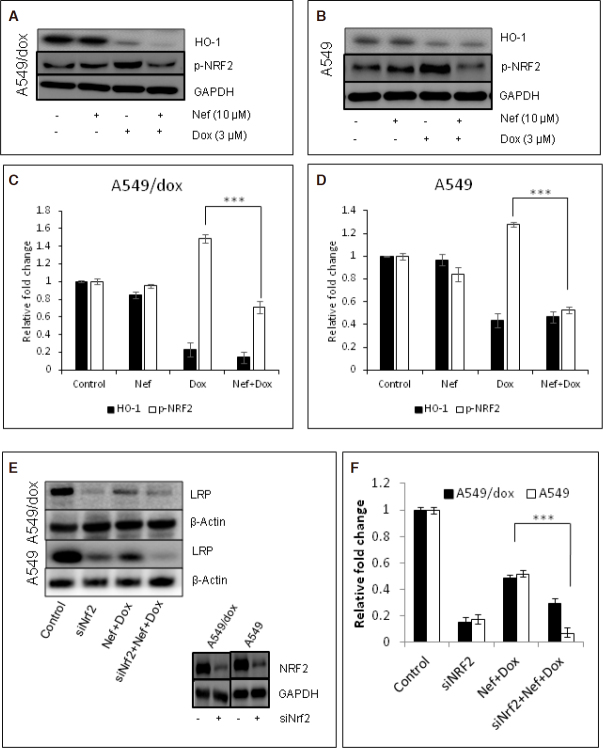
NRF2 regulates LRP expression in lung cancer cells treated with Nef and Dox. A-D: representative western blot analysis of A549 and A549/Dox cells treated with Dox alone, Nef alone and Nef and Dox combination and densitometric analysis are shown for the proteins p-NRF2 and HO-1; E, F: A549 or A549/Dox cells were knocked out for NRF2 using siNRf2 and treated with Dox in combination with Nef. Cell lysates were analyzed for LRP expression and a representative image and densitometric analysis were shown. GAPDH served as internal control for western blots. The results in the graph shown are mean ± SEM and *** represents *P* < 0.01 which are 3 separate experiments performed in triplicate. Nef: Neferine; Dox: Doxorubicin; NRF2: nuclear factor erythroid-derived 2-like 2; LRP: lung resistance protein; GAPDH: Glyceraldehyde 3-phosphate dehydrogenase

We further evaluated the effect of Nef and Dox combination on NRF2 which is a major antioxidant response protein and has implicated in drug resistance. It has been shown that acquisition of Dox resistance accompanies NRF2 overexpression^[46]^. The p-NRF2 and HO-1 (one of the cytoprotective protein regulated by NRF2) expression were significantly reduced by Nef in the cells treated with Dox [Figure 6A-D]. To confirm whether NRF2 was involved in the chemo resistance, the lung cancer cells were transfected with NRF2 siRNA to knockdown the NRF2 expression. In the absence of NRF2 both A549 and A549/Dox cells were sensitive to Dox than the respective controls. Hence we were interested to know whether NRF2 knockdown had any correlation with LRP expression. As anticipated, LRP expression levels were significantly reduced after NRF2 knock down [Figure 6E and F]. These results clearly indicate the significance of NRF2 and LRP in Nef mediated reversal of drug resistance.

### Effect of Nef and Dox combination on multicellular lung tumor spheroids

**Figure 7:**
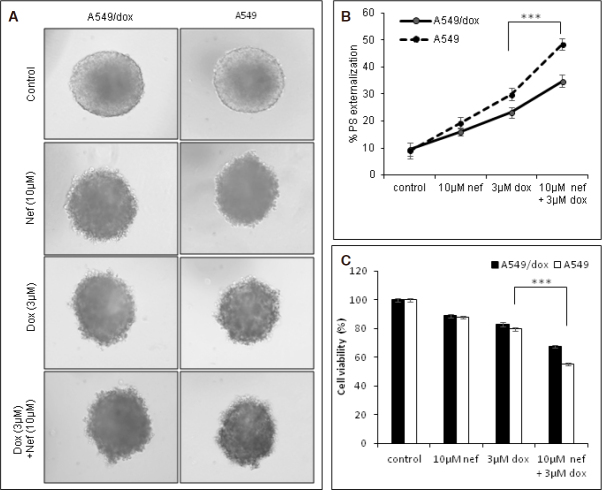
Nef and Dox combination induces apoptosis in 3D multicellular spheroids of lung cancer cells. Multicellular spheroids of A549 and A549/Dox cells were generated and treated with Nef/Dox alone or in combination. A: light microscopic images of the spheroids after treatment period (100× magnification); B: line graph showing the percentage (%) of phosphatidyserine externalization using flow cytometric analysis; C: bar graph showing the cell viability of multicellular spheroids. The results in the graph shown are mean ± SEM and *** represents *P* < 0.01 which are 3 separate experiments performed in triplicate. Nef: Neferine; Dox: Doxorubicin; PS: phosphatidyserine

To extrapolate our results to 3D model system, multicellular tumor spheroids were used for the study. Initially, the effect of Nef and/or Dox treatment on spheroids size was analyzed microscopically. The spheroid shrinkage was significant with the combination regimen than the individual treatment [Figure 7A]. Furthermore, the cell viability of the spheroids was determined. Nef cotreatment with Dox could effectively increase the %PS externalization and reduce the cell viability than the Dox alone or Nef alone treated tumor spheroid groups [Figure 7B and C].

## Discussion

Multidrug resistance management is a major challenge associated with currently used chemotherapeutics in clinic for cancer treatment. Investigating novel compounds for potential mechanisms to overcome resistance, enhancing therapeutic effect and minimizing chemotherapy associated side effects continues to be an area of intense research to improve survival and the quality of life of patients with cancer. Dox is among the most effective anticancer drugs known, and widely used as a first line treatment for several cancers, including lung cancer. However, repeated treatment with Dox leads to the development of drug resistance and dose-limiting cardiotoxicity^[6]^. Various phytochemicals have been used to overcome Dox related resistance and cardiotoxicity, and to improve treatment efficacy. Nef is an alkaloid from lotus, previously reported to possess anticancer and cardioprotective effects, and to enhance the efficacy of chemotherapeutics^[30,36,38,41,42]^.

We hypothesized that Nef co-administered with Dox would overcome Dox resistance and enhance its therapeutic effect without adding to its side effects. In our study, we have used A549 cells which possess point mutation in the Keap1 gene (*G333C*) and loss of heterozygosity at 19p13.2 that leads to the loss of KEAP1 activity and gain of NRF2 function^[44]^. Our data show that Nef in combination with Dox enhances the cell death of A549 and A549/Dox cells monolayers and spheroids. The foremost cause of Dox resistance in lung cancer cells is the over expression of multidrug resistant proteins such as P-gp, LRP, *etc.* that exclude drugs out of the cells^[17]^. Nef significantly increased the intracellular Dox levels in both Dox sensitive and resistant A549 cells. In lung cancer patients, it has been observed that P-gp was mainly found to be coexpressed with LRP, a major-vault protein^[47]^. We already have reported that Nef can reduce P-gp activity in Dox treated lung cancer cells^[35]^. Thus we focused our current study on LRP modulation by Nef and Dox treatment. Efflux of Dox from nucleus by LRP leads to lesser Dox sensitivity of lung cancer cells than breast cancer cells^[17]^. Correlating with this, we could observe higher LRP expression in A549/Dox than A549 cells. Simultaneous treatment of Nef and Dox significantly down regulated Dox induced LRP expression and a concomitant increase in intra-nuclear Dox. LRP knockdown lead to higher Dox accumulation and cell death in cells that received co-treatment suggesting the role of LRP in Nef induced reversal of Dox resistance in lung cancer cells. This data is in parallel with the reports suggesting that the inhibition of LRP overcomes the Dox resistance in lung and ovarian cancer cells, and cisplatin resistance in lung cancer cells^[17,48-50]^.

**Figure 8:**
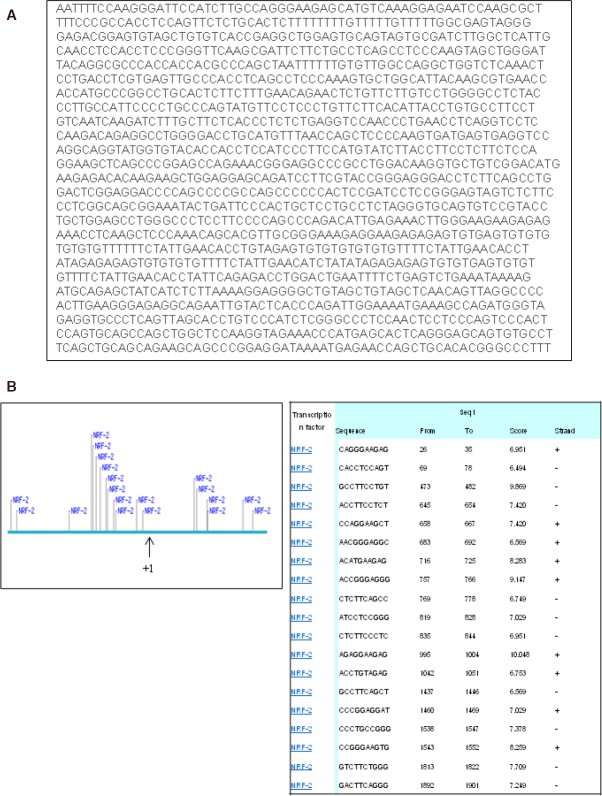
In silico analysis of LRP promoter sequence: 1.5 kb promoter region of LRP region was retrieved from Ensembl database (www.ensembl.org) and putative NRF2 binding sites were predicted (consite.genereg.net) as indicated. A: lung resistance protein or major vault protein - 1.5 kb promoter region; B: putative transcription factor binding sites found along LRP promoter. Sequences highlighted in blue show NRF2 binding sites as predicted by ConSite and +1 indicates the transcriptional start site. NRF2: nuclear factor erythroid-derived 2-like 2; LRP: lung resistance protein

Traditionally considered as cellular byproducts of metabolism, ROS has been recognized as second messengers in signal transduction process influencing growth, survival and overall physiological homeostasis^[51]^. Several studies have illustrated the role of ROS in the action mechanism of Nef^[29,30,35]^. These taken together with our previous work lead to the notion that LRP downregulation might be influenced by ROS^[35]^. We observed that the Nef and Dox cotreatment resulted in ROS hypergeneration and concomitant GSH depletion which induces oxidative stress and leads to cancer cell death. These observations were reinforced by the use of NAC to attenuate ROS levels and desensitize the cytotoxicity of cotreatment. Furthermore, the restoration of LRP levels by NAC, clearly demonstrated the ROS dependency of LRP expression in lung cancer cells after Nef and Dox treatment. The probable association of the transcription factor, NRF2 to drug resistance can be speculated as it is a master regulator of the expression of multiple antioxidant proteins. A549 cells are reported to have higher NRF2 basal expression levels^[52]^. NRF2 was further elevated in A549/Dox cells than their sensitive counterpart. It has been reported that the acquisition of Dox resistance in ovarian cells was accompanied by NRF2 activation^[46]^. It has been reported that the drug transport proteins such as P-glycoprotein (P-gp/ABCB1), multidrug resistance-associated protein (MRP/ABCC) 1/2/3/4/5, and breast cancer resistance protein (BCRP/ABCG2) are positively regulated by NRF2^[20-24]^. As the combination of Nef and Dox lead to oxidative stress regulated LRP expression, it is highly promising to study the effect of NRF2 in LRP expression. As we have speculated, the LRP expression was significantly reduced after NRF2 knockdown in lung cancer cells. The NRF2 knocked down cells after Nef and Dox cotreatment showed further decrease in LRP levels. These results prompted us to study whether the promoter region of the *LRP* gene has NRF2 binding sites. Using ConSite transcription factor binding site prediction tool we performed *in silico* analysis of 1.5 kb promoter region of LRP for the presence of putative NRF2 binding sequences and found a number of such binding sites [Figure 8]. This is the first study to report the relationship between LRP and NRF2 in drug resistance. Further studies are warranted to confirm the transcriptional control of NRF2 on LRP.

Finally, our results of 2D monolayers were validated using 3D models to reciprocate the *in vivo* tumor mass. Cancer cells propagated in 3D culture systems mimics *in vivo* tumors. Our experiment showed that the Nef and Dox combination induced significant reduction in spheroid size and viability compared to individual treatments. In addition, the apoptotic inducing potential of the combination therapy is higher than the Dox treatment alone. Dox induced cardiomyopathy is the major roadblock in cancer treatment in clinic. Our group has previously reported the cardio-protective effect of Nef in cardio myoblasts challenged with Dox and isoproterenol induced myocardial infarction in wistar rats^[41,42]^. It has been shown that inhibition of NRF2 using siRNA could increase the sensitivity of A549 tumors to carboplatin *in vivo*^[53]^. NRF2 inhibitors like brusatol and luteolin were also reported to sensitize A549 tumor xenograft models to cisplatin *in vivo*^[54,55]^. Thus, we presume that neferine with its inhibitory effect on NRF2 could also have similar sensitizing effect in xenograft models. Thus, further *in vivo* studies are warranted for this combination therapy to move forward.

**Figure 9:**
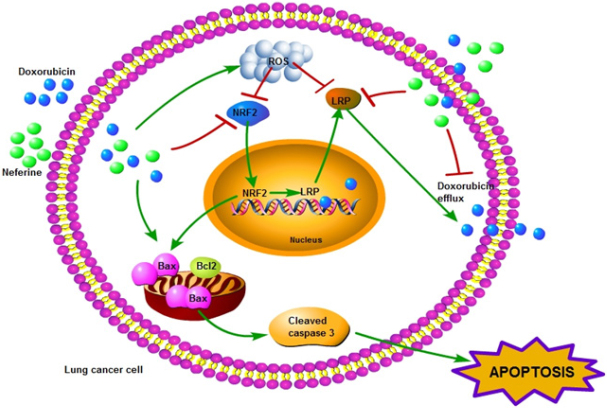
Schematic representation showing the mechanism of action of Nef in overcoming Dox resistance in lung cancer cells. Nef in combination with Dox increases ROS levels which in turn leads to NRF2 mediated LRP downregulation. This effect leads to the increased Dox levels in the lung cancer cells and cell death. Nef: Neferine; Dox: Doxorubicin; NRF2: nuclear factor erythroid-derived 2-like 2; LRP: lung resistance protein; ROS: Reactive oxygen species

In summary, we found that the co-treatment of Dox and Nef in Dox resistant lung cancer cells induced apoptosis in both 2D and 3D models, reversed Dox resistance via NRF2 mediated LRP down regulation. This is the first report on the LRP regulation by NRF2 in lung cancer cells. This study suggests a new molecular mechanism that may contribute to the anticancer activities of Dox-Nef co-therapy in lung cancer cells. These findings provide a rational basis for the application of NRF2 and LRP inhibitors with additional health benefits such as cardio protection would improve the life expectancy of cancer patients. Figure 9 depicts the overall mechanism of action of Nef and Dox combination in reversal of drug resistance in lung cancer cells.

## Declarations

### Copyright

© The Author(s) 2022.

## References

[1] Siegel RL, Miller KD, Jemal A (2018). Cancer statistics, 2018.. CA Cancer J Clin.

[2] Ferlay J, Soerjomataram I, Dikshit R, Eser S, Mathers C (2015). Cancer incidence and mortality worldwide: sources, methods and major patterns in Globocan 2012.. Int J Cancer.

[3] Chan BA, Hughes BGM (2015). Targeted therapy for non-small cell lung cancer: current standards and the promise of the future.. Transl Lung Cancer Res.

[4] Gewirtz DA (1999). A critical evaluation of the mechanisms of action proposed for the antitumor effects of the anthracycline antibiotics adriamycin and daunorubicin.. Biochem Pharmacol.

[5] Weiss RB (1992). The anthracyclines: will we ever find a better doxorubicin?. Semin Oncol.

[6] Arafa el-SA, Zhu Q, Shah ZI, Wani G, Barakat BM (2011). Thymoquinone up-regulates PTEN expression and induces apoptosis in doxorubicin-resistant human breast cancer cells.. Mutat Res.

[7] Mi J, Zhang X, Rabbani ZN, Liu Y, Reddy SK (2008). RNA aptamer-targeted inhibition of NF-kappa B suppresses non-small cell lung cancer resistance to doxorubicin.. Mol Ther.

[8] Choi CH (2005). ABC transporters as multidrug resistance mechanisms and the development of chemosensitizers for their reversal.. Cancer Cell Int.

[9] Salehan MR, Morse HR (2013). DNA damage repair and tolerance: a role in chemotherapeutic drug resistance.. Br J Biomed Sci.

[10] Wilson TR, Johnston PG, Longley DB (2009). Anti-apoptotic mechanisms of drug resistance in cancer.. Curr Cancer Drug Targets.

[11] Lee C, Raffaghello L, Longo VD (2012). Starvation, detoxification, and multidrug resistance in cancer therapy.. Drug Resist Updat.

[12] Sissung TM, Baum CE, Kirkland CT, Gao R, Gardner ER (2010). Pharmacogenetics of membrane transporters: an update on current approaches.. Mol Biotechnol.

[13] Laurençot CM, Scheffer GL, Scheper RJ, Shoemaker RH (1997). Increased LRP mRNA expression is associated with the MDR phenotype in intrinsically resistant human cancer cell lines.. Int J Cancer.

[14] Berger W, Elbling L, Micksche M (2000). Expression of the major vault protein LRP in human non-small-cell lung cancer cells: activation by short-term exposure to antineoplastic drugs.. Int J Cancer.

[15] Scheffer GL, Schroeijers AB, Izquierdo MA, Wiemer EA, Scheper RJ (2000). Lung resistance-related protein/major vault protein and vaults in multidrug-resistant cancer.. Curr Opin Oncol.

[16] Scheper RJ, Broxterman HJ, Scheffer GL, Kaaijk P, Dalton WS (1993). Overexpression of a M(r) 110,000 vesicular protein in non-P-glycoprotein-mediated multidrug resistance.. Cancer Res.

[17] Meschini S, Marra M, Calcabrini A, Monti E, Gariboldi M (2002). Role of the lung resistance-related protein (LRP) in the drug sensitivity of cultured tumor cells.. Toxicol In Vitro.

[18] Scheffer GL, Wijngaard PLJ, Flens MJ, Izquierdo MA, Slovak ML (1995). The drug resistance-related protein LRP is the human major vault protein.. Nat Med.

[19] Singh A, Boldin-Adamsky S, Thimmulappa RK, Rath SK, Ashush H (2008). Mediated silencing of nuclear factor erythroid-2–related factor 2 gene expression in non–small cell lung cancer inhibits tumor growth and increases efficacy of chemotherapy.. Cancer Res.

[20] Bachas S, Eginton C, Gunio D, Wade H (2011). Structural contributions to multidrug recognition in the multidrug resistance (MDR) gene regulator, BmrR.. Proc Natl Acad Sci U S A.

[21] Ji L, Li H, Gao P, Shang G, Zhang DD (2013). Nrf2 pathway regulates multidrug-resistance-associated protein 1 in small cell lung cancer.. PLoS One.

[22] Singh A, Wu H, Zhang P, Happel C, Ma J (2010). Expression of ABCG2 (BCRP) is regulated by Nrf2 in cancer cells that confers side population and chemoresistance phenotype.. Mol Cancer Ther.

[23] Stockel B, Konig J, Nies AT, Cui Y, Brom M (2000). Characterization of the 5’-flanking region of the human multidrug resistance protein 2 (MRP2) gene and its regulation in comparison withthe multidrug resistance protein 3 (MRP3) gene.. Eur J Biochem.

[24] Xu S, Weerachayaphorn J, Cai SY, Soroka CJ, Boyer JL (2010). Aryl hydrocarbon receptor and NF-E2-related factor 2 are key regulators of human MRP4 expression.. Am J Physiol Gastrointest Liver Physiol.

[25] Wang X, Campos CR, Peart JC, Smith LK, Boni JL (2014). Nrf2 Upregulates ATP binding cassette transporter expression and activity at the blood-brain and blood–spinal cord barriers.. J Neurosci.

[26] Endres CJ, Hsiao P, Chung FS, Unadkat JD (2006). The role of transporters in drug interactions.. Eur J Pharm Sci.

[27] Xue F, Liu Z, Xu J, Xu X, Chen X (2019). Neferine inhibits growth and migration of gastrointestinal stromal tumor cell line GIST-T1 by up-regulation of miR-449a.. Biomed Pharmacother.

[28] Li XC, Tong GX, Zhang Y, Liu SX, Jin QH (2010). Neferine inhibits angiotensin II-stimulated proliferation in vascular smooth muscle cells through heme oxygenase-1.. Acta Pharmacol Sin.

[29] Poornima P, Quency RS, Padma VV (2013). Neferine induces reactive oxygen species mediated intrinsic pathway of apoptosis in HepG2 cells.. Food Chem.

[30] Poornima P, Weng CF, Padma VV (2014). Neferine, an alkaloid from lotus seed embryo, inhibits human lung cancer cell growth by MAPK activation and cell cycle arrest.. Biofactors.

[31] Zhang X, Liu Z, Xu B, Sun Z, Gong Y (2012). Neferine, an alkaloid ingredient in lotus seed embryo, inhibits proliferation of human osteosarcoma cells by promoting p38 MAPK-mediated p21 stabilization.. Eur J Pharmacol.

[32] Cao JG, Tang XQ, Shi SH (2004). Multidrug resistance reversal in human gastric carcinoma cells by neferine.. World J Gastroenterol.

[33] Ai XH, Tang XQ, Liu YP, Liu HQ, Dong L (2007). Effect of neferine on adriamycin-resistance of thermotolerant hepatocarcinoma cell line HepG2/thermotolerance.. Ai Zheng.

[34] Tang XQ, Cao JG (2001). Enhancement of cytotoxicity of anticancer drugs in vitro by neferine in MCF-7 cells.. Chin J Modern Appl Pharmacy.

[35] Poornima P, Kumar VB, Weng CF, Padma VV (2014). Doxorubicin induced apoptosis was potentiated by neferine in human lung adenocarcima, A549 cells.. Food Chem Toxicol.

[36] Sivalingam KS, Paramasivan P, Weng CF, Viswanadha VP (2017). Neferine potentiates the antitumor effect of cisplatin in human lung adenocarcinoma cells via a mitochondria-mediated apoptosis pathway.. J Cell Biochem.

[37] Deng G, Zeng S, Ma J, Zhang Y, Qu Y (2017). The anti-tumor activities of Neferine on cell invasion and oxaliplatin sensitivity regulated by EMT via Snail signaling in hepatocellular carcinoma.. Sci Rep.

[38] Baskaran R, Poornima P, Huang CY, Padma VV (2016). Neferine prevents NF-kappaB translocation and protects muscle cells from oxidative stress and apoptosis induced by hypoxia.. Biofactors.

[39] Baskaran R, Poornima P, Priya LB, Huang CY, Padma VV (2016). Neferine prevents autophagy induced by hypoxia through activation of Akt/mTOR pathway and Nrf2 in muscle cells.. Biomed Pharmacother.

[40] Baskaran R, Priya LB, Kalaiselvi P, Poornima P, Huang CY (2017). Neferine from Nelumbo nucifera modulates oxidative stress and cytokines production during hypoxia in human peripheral blood mononuclear cells.. Biomed Pharmacother.

[41] Lalitha G, Poornima P, Archanah A, Padma VV (2013). Protective effect of neferine against isoproterenol-induced cardiac toxicity.. Cardiovasc Toxicol.

[42] Priya LB, Baskaran R, Huang CY, Padma VV (2017). Neferine ameliorates cardiomyoblast apoptosis induced by doxorubicin: possible role in modulating NADPH oxidase/ROS-mediated NFκB redox signaling cascade.. Sci Rep.

[43] Pereira-Caro G, Mateos R, Sarria B, Cert R, Goya L (2012). Hydroxytyrosyl acetate contributes to the protective effects against oxidative stress of virgin olive oil.. Food Chem.

[44] Singh A, Misra V, Thimmulappa RK, Lee H, Ames S (2006). Dysfunctional KEAP1-NRF2 interaction in non-small-cell lung cancer.. PLoS Med.

[45] Kitazono M, Sumizawa T, Takebayashi Y, Chen ZS, Furukawa T (1999). Multidrug resistance and the lung resistance-related protein in human colon carcinoma.. J Natl Cancer Inst.

[46] Shim GS, Manandhar S, Shin DH, Kim TH, Kwak MK (2009). Acquisition of doxorubicin resistance in ovarian carcinoma cells accompanies activation of the NRF2 pathway.. Free Radic Biol Med.

[47] Lario AP, García CB, Elizondo ME, Lobo C (2007). Expression of proteins associated with multidrug resistance to chemotherapy in lung cancer.. Arch Bronconeumol.

[48] Przystupski D, Michel O, Rossowska J, Kwiatkowski S, Saczko J (2019). The modulatory effect of green tea catechin on drug resistance in human ovarian cancer cells.. Med Chem Res.

[49] Chen YL, Yang TY, Wu CL, Chen KC, Hsu SL (2016). Mechanisms underlying lung resistance-related protein (LRP)-mediated doxorubicin resistance of non-small cell lung cancer cells.. Chinese J Physiol.

[50] Zhang W, Zhou H, Yu Y, Li J, Li H (2016). Combination of gambogic acid with cisplatin enhances the antitumor effects on cisplatin-resistant lung cancer cells by downregulating MRP2 and LRP expression.. OncoTargets Ther.

[51] Forman HJ, Maiorino M, Ursini F (2010). Signaling functions of reactive oxygen species.. Biochemistry.

[52] Homma S, Ishii Y, Morishima Y, Yamadori T, Matsuno Y (2009). Nrf2 enhances cell proliferation and resistance to anticancer drugs in human lung cancer.. Clin Cancer Res.

[53] Singh A, Boldin-Adamsky S, Thimmulappa RK, Rath SK, Ashush H (2008). RNAi-mediated silencing of nuclear factor erythroid-2-related factor 2 gene expression in non-small cell lung cancer inhibits tumor growth and increases efficacy of chemotherapy.. Cancer Res.

[54] Chian S, Thapa R, Chi Z, Wang XJ, Tang X (2014). Luteolin inhibits the Nrf2 signaling pathway and tumor growth in vivo.. Biochem Biophys Res Commun.

[55] Tao S, Wang S, Moghaddam SJ, Ooi A, Chapman E (2014). Oncogenic KRAS confers chemoresistance by upregulating NRF2.. Cancer Res.

