# Supplementary material for: Reversal of doxorubicin resistance in lung cancer cells by neferine is explained by nuclear factor erythroid-derived 2-like 2 mediated lung resistance protein down regulation
Source: Cancer Drug Resist. 2020 Apr 17;3(3):647–65. doi: 10.20517/cdr.2019.115 (PMC8992493; doi:10.20517/cdr.2019.115)
Supplement: Supplementary file 1 [file cdr-3-647-SupplementaryMaterials.pdf]

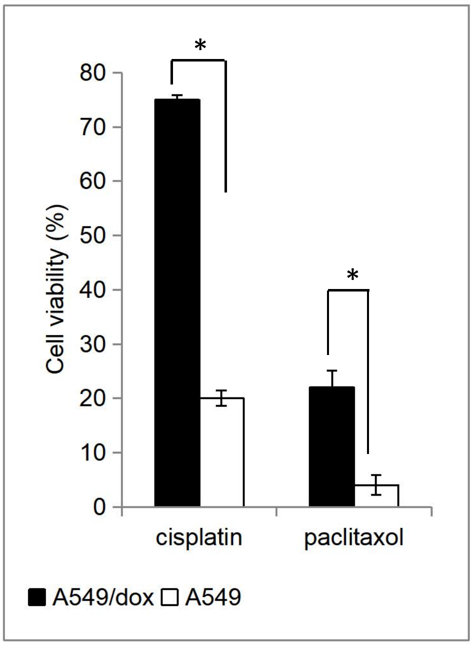

a. A549 dox cells showed cross resistance to paclitaxel and cisplatin

Results shown are Mean± SEM, which are three separate experiments performed in triplicate. Significance is shown at $P<0.01$ (One way ANOVA followed by Tukey's multiple comparison test).

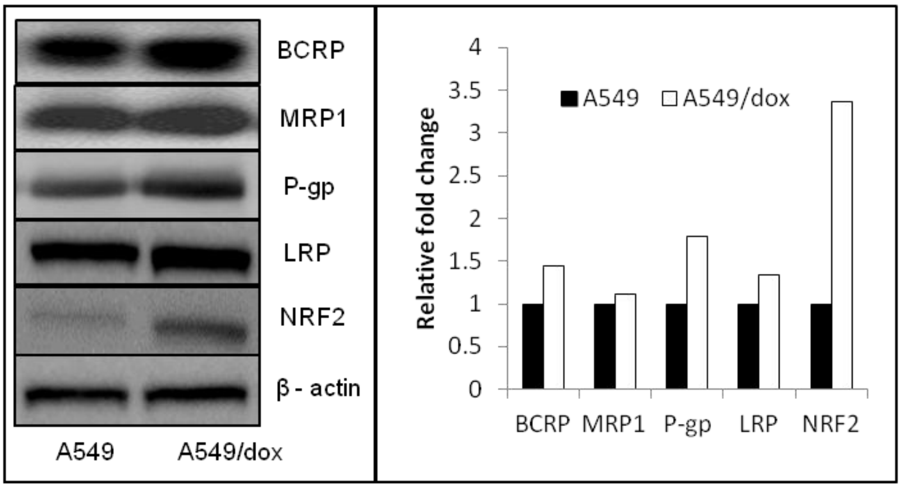

b. The levels of BCRP, MRP1, P-gp, LRP and NRF2 in A549 and A549/dox cells. A549 and A549 cells were analysed for BCRP, MRP1, P-gp, LRP and NRF2 expression, representative western blot images and densitometric analysis are shown.

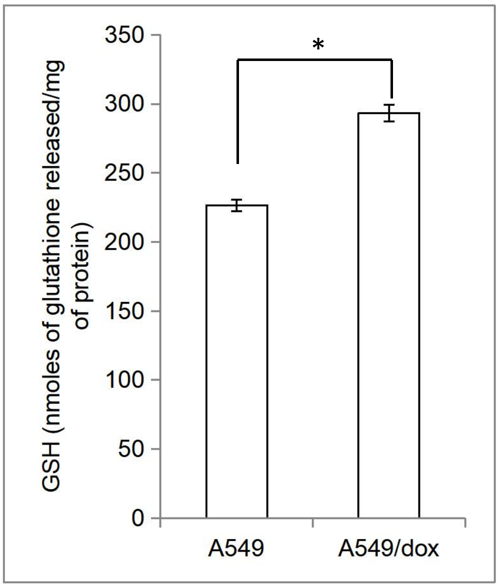

c. The intracellular GSH levels in A549 and A549/dox cells

Results shown are Mean± SEM, which are three separate experiments performed in triplicate. Significance is shown at P<0.01 (One way ANOVA followed by Tukey's multiple comparison test).

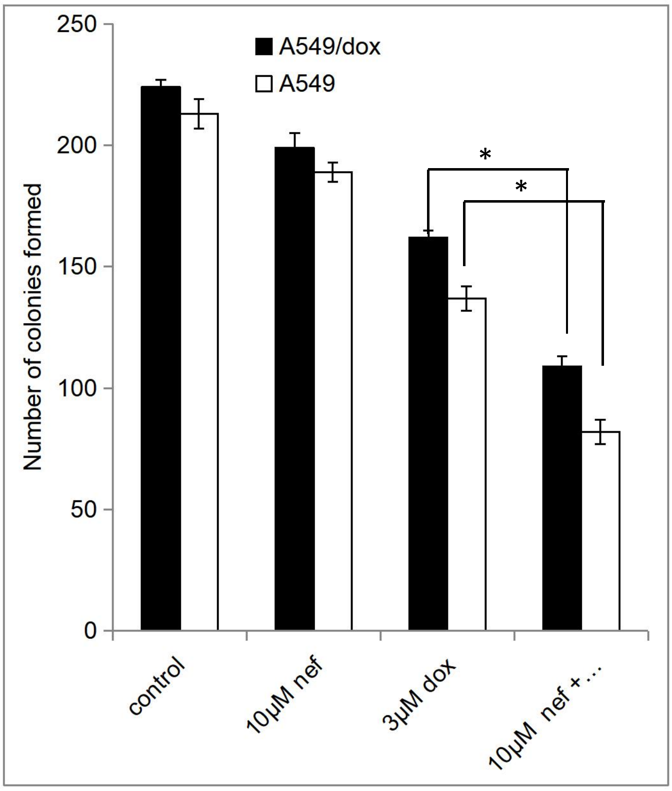

d. Number colonies formed by A549 and A549/dox cells

Results shown are Mean± SEM, which are three separate experiments performed in triplicate. Significance is shown at P<0.01 (One way ANOVA followed by Tukey's multiple comparison test).

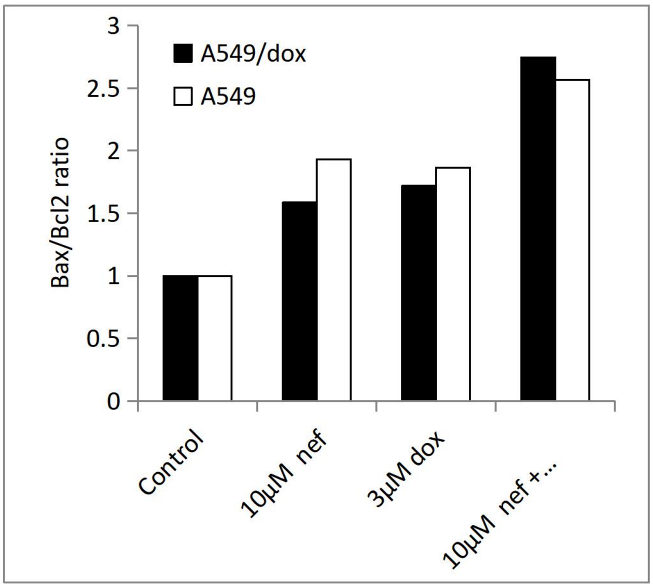

e. Bax/Bcl2 ratio in A549 and A549/dox cells

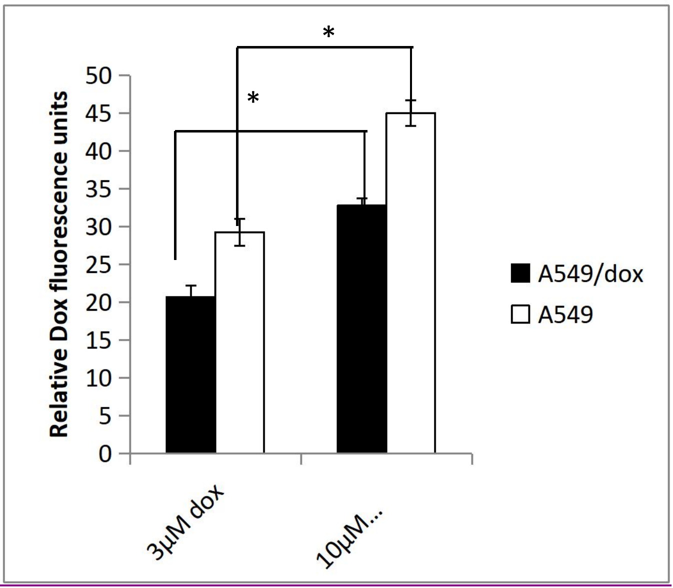

e. Intracellular DOX accumulation was potentiated by neferine. Bar chart shows the intensity of the Dox fluorescence quantified using Image J. Results shown are Mean± SEM, of three separate measurements performed. Significance is shown at P<0.01 (One way ANOVA followed by Tukey's multiple comparison test).
